# Exploiting microglial and peripheral immune cell crosstalk to treat Alzheimer’s disease

**DOI:** 10.1186/s12974-019-1453-0

**Published:** 2019-04-05

**Authors:** Dawling A. Dionisio-Santos, John A. Olschowka, M. Kerry O’Banion

**Affiliations:** Department of Neuroscience, Del Monte Institute for Neuroscience, University of Rochester School of Medicine and Dentistry, 601 Elmwood Avenue, Box 603, Rochester, NY 14642 USA

**Keywords:** Alzheimer’s disease, Microglia, Cytokines, Innate immunity, Adaptive immunity, Therapeutics

## Abstract

Neuroinflammation is considered one of the cardinal features of Alzheimer’s disease (AD). Neuritic plaques composed of amyloid β and neurofibrillary tangle-laden neurons are surrounded by reactive astrocytes and microglia. Exposure of microglia, the resident myeloid cell of the CNS, to amyloid β causes these cells to acquire an inflammatory phenotype. While these reactive microglia are important to contain and phagocytose amyloid plaques, their activated phenotype impacts CNS homeostasis. In rodent models, increased neuroinflammation promoted by overexpression of proinflammatory cytokines can cause an increase in hyperphosphorylated tau and a decrease in hippocampal function. The peripheral immune system can also play a detrimental or beneficial role in CNS inflammation. Systemic inflammation can increase the risk of developing AD dementia, and chemokines released directly by microglia or indirectly by endothelial cells can attract monocytes and T lymphocytes to the CNS. These peripheral immune cells can aid in amyloid β clearance or modulate microglia responses, depending on the cell type. As such, several groups have targeted the peripheral immune system to modulate chronic neuroinflammation. In this review, we focus on the interplay of immunomodulating factors and cell types that are being investigated as possible therapeutic targets for the treatment or prevention of AD.

## Introduction

Neuroinflammation is considered one of the cardinal features of Alzheimer’s disease (AD). Early observations of neuritic plaques composed of amyloid β and neurofibrillary tangle-laden neurons surrounded by reactive astrocytes and microglia [1–4], as well as evidence that microglia exposed to amyloid β release proinflammatory factors such as interleukin 1 beta (IL-1β) and tumor necrosis factor alpha (TNFα) [5–7] that in turn could modify AD pathology [8–12], led to hypotheses positing chronic neuroinflammation as a driving feature of AD [13]. Subsequent investigations revealed a more complex picture, with increased recognition of factors and cell types involved in neuroinflammation, including the influence of peripheral immune mediators and their effects on AD pathology and associated cognitive function in animal models. These findings related to the role of neuroinflammation in AD have been reviewed in multiple articles [14–18]. In this review, we focus on the interplay between factors and cell types associated with neuroinflammation, including cytokines, microglia, and peripheral immune mediators, as well as some clinical studies, to describe how modulation of such processes might be exploited for prevention or treatment of AD.

### Cytokines in AD and effects on pathology

Concentration of various proinflammatory cytokines including IL-1β, IL-6, IL-12, IL-18, and TNFα and anti-inflammatory cytokines such as interleukin-1 receptor antagonist (IL-1RA) and IL-10 have been found to increase in cerebrospinal fluid (CSF) of AD patients, pointing to an immune disturbance [19–22]. Whether production of these cytokines initiates AD or results from neurodegeneration and neuritic plaque deposition has not been conclusively demonstrated in humans. Murine models of AD have been useful in testing the effects of various cytokines on amyloid β and tau pathology. In general, most studies have found that overexpression of pro-inflammatory cytokines leads to a reduction of amyloid β plaque, possibly through microglial activity [10, 23–25]. Nonetheless, proinflammatory factors, such as IL-1β, IL-6, and TNFα, have also been shown to worsen tau hyperphosphorylation and thus could contribute to neurofibrillary tangle formation [8, 11, 26]. In addition, genetic studies have demonstrated that polymorphisms in various cytokines, such as IL-1β, IL-6, IL-12, IL-18, and TNFα, and cytokine receptors, such as the IL-1 receptor accessory protein, are associated with an increased risk of developing AD [27–29]. While the risk of developing AD due to these mutations is modest, they can hasten disease progression when concomitantly present with variants of apolipoprotein E (APOE) that are associated with late-onset AD [27]. Thus, cytokine production could be a potential therapeutic target to slow the progression of sporadic AD.

There is a possibility that AD progression is partially due to a dysregulation of sterile inflammation, such as the one that is induced in the brain with amyloid β accumulation. One of the components of the sterile inflammatory response is the formation of a multiprotein complex called the inflammasome, which is critical to convert precursors of IL-1β and IL-18 to the biologically active cytokines [30]. Fibrillar amyloid β has been shown to bind NOD-like receptor protein 3 (NLRP3), one of the pattern recognition receptors that regulates inflammasome formation in microglia and induces IL-1β production [31]. Deletion of NLRP3 ameliorated amyloid β pathology and improved cognitive impairment in APP/PS1 mice [32], perhaps by reducing cross-seeding of amyloid β [33]. Administration of NLRP3 inhibitors have decreased cerebral amyloid β and improved cognitive function in amyloidogenic models making inflammasome formation a promising therapeutic target [34–36]. Further discussion of the role of inflammasomes in AD and neurodegeneration can be found in recent reviews [37–39].

In addition to the proinflammatory changes that occur with aging and AD, there is evidence to suggest a dysregulation in anti-inflammatory pathways. Immunohistochemical studies have revealed that the neurotrophic factor TGF-β2 is localized in reactive astrocytes and microglia surrounding neuritic plaques [40]. In addition, increased concentrations of TGF-β2 have been detected in the AD patient CSF [41]. Expression of this cytokine in reactive glia may represent an attempt to curb the inflammatory response initiated by amyloid β accumulation. In AD patients, neuronal TGF-β2 signaling may be blunted due to decreased expression of TGFβRII that is present even in prodromal stages of AD [42]. Like proinflammatory cytokine modulation, overexpression of anti-inflammatory cytokines, such as IL-4, IL-13, and TGF-β1, has been shown to decrease amyloid beta plaque [43–45]. Stimulation with anti-inflammatory cytokines alternatively activates microglia, upregulating markers such as arginase 1 and YM1. One group demonstrated that hippocampal arginase 1 overexpression in rTg4510 tau transgenic mice led to a decrease in tau phosphorylation [46].

### The role and modulation of microglia in AD

Microglia are the resident macrophages of the central nervous system. During homeostatic conditions, microglia survey the brain and participate in the maintenance and pruning of synapses [47, 48]. However, like macrophages in other tissues, microglia become activated when they encounter an offending stimulus or pathogen. In the context of AD, neuritic plaques are typically surrounded by extensive astrogliosis and microgliosis. Microglia surrounding plaques acquire an amoeboid morphology, which is associated with high phagocytic activity. Several lines of research demonstrated that amyloid β binds toll-like receptor 4 and its co-activator CD14, polarizing microglia towards an inflammatory phenotype which includes the release of proinflammatory cytokines like IL-1β, IL-6, and TNFα, as well as the chemokine CCL2 [6, 7, 49, 50]. As described above, these cytokines aid in the activation of other microglia to increase their phagocytic capabilities.

Amyloid β can exist as soluble oligomers or insoluble fibrils of the proteolytic products Aβ 1-40 or 1-42. While attention has largely focused on studying the effect of insoluble fibrils on neurons and glia, amyloid β oligomers are also thought to contribute to disease pathology. Aβ 1-42 oligomers, more so that Aβ 1-40 oligomers, have been shown to be neurotoxic and can elicit acute microgliosis and impairment in long-term potentiation when injected into the brain of wild-type mice [51, 52]. Furthermore, microglia are capable of phagocytizing all forms of amyloid β, including soluble oligomers [53]. Interestingly, the response of microglia to amyloid β is different depending on whether it exists as soluble oligomers or fibrillar plaques. In an in vitro study, oligomers stimulated increased levels of phagocytosis markers including activated Lyn and Syk kinase as well as p38MAPK compared to fibrils [54]. In addition, microglia treated with oligomers increased the production of cytokines IL-6 and CCL2 when compared to fibrils [54, 55]. Some investigators have considered amyloid β plaques to be a relatively benign aggregation of amyloid β that microglia contain to protect the brain from the more neurotoxic oligomers [56–58].

Microglia employ several strategies in order to contain and clear amyloid β pathology. Microglia surround amyloid β plaques in an effort to isolate them from the rest of the brain parenchyma [57]. In addition, microglia can release enzymes that aid in the degradation of amyloid β plaques. Complement activation is also important for amyloid β phagocytosis. There is increased C1 surrounding neuritic plaques in AD patients’ tissue samples [59]. In vitro studies have shown that human C1q is able to bind amyloid β and thus could initiate the complement cascade, allowing amyloid β plaques to be opsonized and facilitate phagocytosis by microglia [60]. Interferon γ-induced C3 upregulation in an amyloidogenic mouse model caused a decrease in amyloid β plaque size and number [61], and inhibition of C3 with sCrry or C3 knockout caused increased plaque deposition [62, 63]. However, upregulation of C1q and C3 also resulted in increased phagocytosis of synapses by microglia and appeared to be necessary for cognitive dysfunction in amyloidogenic models, indicating that this clearance mechanism might be a double-edged sword [64, 65]. C1q tags pathological tau-laden synapses, leading to microglial engulfment and synapse loss [66]. In addition, deletion of the C3a receptor in PS19 tau mice decreased neuroinflammation, attenuated tau pathology, and ameliorated disease-associated microglia and neurotoxic astrocyte signature [67].

The ability of microglia to surround and phagocytize amyloid β plaques depends on the microglia-expressed gene *Trem2*. This gene encodes a protein called triggering receptor expressed on myeloid cells 2 that is expressed by innate immune cells. Among the functions of TREM2 are regulation of immune responses, expression of constitutive cytokines, and microglial response to neuronal injury [68, 69]. Mutations in TREM2 have been identified as a risk factor for the development of late-onset AD [70–72]. Mice deficient in TREM2 tend to have diffuse, irregularly-shaped plaques with multiple hotspots of Aβ 1-42 polymerization [73, 74]. Furthermore, these plaques were associated with increased neurodegeneration when compared to TREM2 sufficient mice [74]. In addition, TREM2 deficiency induced inflammation shown by an increase in IL-1β secretion and Iba1 staining in microglia [73–75]. Possibly because of the increase in neuroinflammation, TREM2 deficiency also exacerbated tau hyperphosphorylation in a tauopathy murine model [76]. These findings suggest that TREM2 activity regulates the inflammatory response by microglia and thus might be a target for modulation of their activation.

Microglia can also acquire an anti-inflammatory, neuroprotective phenotype in the context of AD. Evidence of the anti-inflammatory mechanisms in AD comes from studies that manipulated the neuroinflammatory environment in AD mouse models. Induction of neuroinflammation through acute intra-cortical lipopolysaccharide (LPS) injection or IL-1β overexpression in the hippocampus not only increased the number of proinflammatory nitric oxide synthase (iNOS) positive microglia, but also induced the expression of anti-inflammatory marker arginase 1 in some microglia [26, 45]. Interestingly, arginase 1-positive cells appeared to be better phagocytes of amyloid β when compared to iNOS-positive cells and IL-1β-dependent plaque clearance was inhibited using antibodies against the IL-4 receptor (IL-4Rα) [45]. Intrahippocampal injection of anti-inflammatory cytokines IL-4 and IL-13 also induced arginase 1 expression and reduced pathology in amyloidogenic murine models [44, 45]. Arginase 1 overexpression alone was able to cause a reduction in tau hyperphosphorylation, while arginase inhibition led to an increase in tau pathology [46]. What predisposes some microglia to move along the spectrum of proinflammatory and anti-inflammatory phenotypes in the context of AD is currently unknown. In spinal cord injury, inflammation induces expression of IL-4Rα and IL-4 mRNA [77]. Interestingly, IL-4 signaling correlates with not only an increase in arginase 1 signaling but also an increase in IL-1β and CCL2 expression that is not present in a microglia-specific IL-4Rα knockout mouse [77]. While initial studies sought to categorize these cells into either proinflammatory M1 cells or anti-inflammatory M2 cells, the reality is that microglial phenotypes show greater diversity during development and pathology [78–81], and the use of this simplistic nomenclature has been discouraged [82, 83].

Recently, careful attention has been given to describe microglia phenotypes. Advances such as single-cell RNA sequencing have revealed that immune cells in the brain comprise a more heterogeneous population than previously thought [84, 85]. In the healthy brain, homeostatic microglia are tightly regulated by TGF-β1 [86, 87]. Deletion of this gene leads to a downregulation of the homeostatic signature of microglia, which is characterized by the expression of *P2ry12*, *Tmem119*, *Sall1*, and *Mertk*, among other genes. Phenotypically, mice with absent TGF-β1 developed late-onset motor deficits and have synaptic plasticity and glutamate recycling abnormalities [86].

This TGF-β1-dependent state is also downregulated during neurodegenerative processes to give rise to a more phagocytic, inflammatory phenotype termed disease-associated microglia (DAM) or the microglia of neurodegenerative disease [88, 89]. In addition to a loss of homeostatic markers, the DAM phenotype has been characterized by an upregulation of phagocytic genes including *Trem2*, *Apoe*, *Axl*, *Lpl*, and *Clec7a*. DAM activation occurs in a stepwise manner with a TREM2-independent transitional stage followed by a TREM2-dependent state [88]. In AD, this program is specifically adopted by microglia surrounding amyloid β plaques in both humans and mouse models [89]. Interestingly, this program deviates from the M1 and M2 phenotype as DAM express both classic M2 markers, such as arginase 1 and YM1, and proinflammatory genes such as *Il1b*, *PTGS2*, *Ccl2*, *Ccl5*, *Tspo*, *Msr1*, and *Cebpb* [89]. In contrast, microglia stimulated with LPS or IFN γ, which are known inducers of the M1 phenotype, have low expression of *Apoe*, which is one of the most significantly upregulated genes in DAM cells [89].

**Fig. 1:**
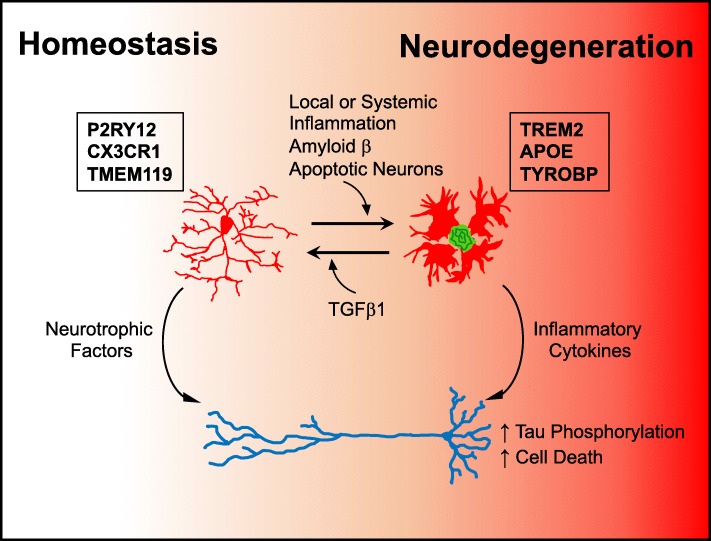
Factors impacting microglial phenotype in the context of homeostasis and neurodegeneration. Adult microglia are characterized by the expression of *P2ry12*, *Cx3cr1*, and *Tmem119*, among others. In addition to their role in immune surveillance, microglia participate in central nervous system homeostasis and are known to release neurotrophic factors that promote neuronal health. During Alzheimer’s disease pathogenesis, microglia are activated by amyloid β through binding of TLR4 and, in a TREM2 dependent manner, downregulate expression of homeostatic genes and upregulate *Apoe*, *Tyrobp*, *Trem2*, and other genes, adopting the disease-associated microglia (DAM) program. DAM are effective at containing and phagocytosing amyloid β plaques. At the same time, DAM cease their neuroprotective functions and release neuroinflammatory factors such as IL-1β and TNFα that are directly damaging to neurons and lead to tau phosphorylation and subsequent cognitive decline. In addition to local factors, the peripheral immune system can modify these responses and induce the DAM program, indicating that this is a potential target through which the homeostatic and inflammatory balance can be modulated and perhaps used to promote beneficial outcomes in AD

The phagocytic nature of the DAM program might be beneficial for the clearance or containment of proteinaceous deposits such as amyloid β. Similarly to the peripheral immune system, microglia have mechanisms to counterbalance this activation and return to homeostasis, including binding of CX3CR1 and CD200R to their respective ligands [90]. However, these immune checkpoints are downregulated during the course of DAM activation [88, 89]. Thus, with increased accumulation of amyloid β plaques, microglia may become dysregulated and unable to resume their neuroprotective functions, which include promoting neuronal health and secreting immunosuppressive factors, such as TGF-β1, which have been shown to prevent inflammatory cascades from persisting [91]. Furthermore, these phagocytic microglia may also partake in excessive synaptic pruning, contributing to memory loss [64]. Indeed, TREM2 deletion has been associated with an increase in the proportion of homeostatic microglia and a reduction in amyloid β plaques at early stages of amyloidogenesis [83, 89, 92]. Still, future studies evaluating the protective functions of DAM and the balance between pathological response and homeostasis offer a promising route for the development of comprehensive AD treatments that affect multiple disease hallmarks. In Fig. 1, we briefly summarize the role of microglia during homeostasis and AD neurodegeneration. Furthermore, microglial responses can be modulated by systemic inflammation and peripheral immune cells, which will be discussed later in this review.

In addition to changes during disease progression, microglia may also become dysfunctional and lose the capacity to return to their neuroprotective functions with aging [93]. Among the changes that occur in the aging brain are an increase in activation elements of microglia including toll-like receptor signaling and complement activation. In addition, there is a decrease of factors that curtail neuroinflammation including IL-10, fractalkine, and toll-interacting protein [20]. Careful morphological analysis of microglia in tissue from advanced AD patients revealed extensive cytorrhexis or fragmentation of microglia rather than amoeboid microglia [93]. Thus, late in disease, microglia might become unable to respond and control amyloid β pathology in addition to not being able to carry out their neuroprotective functions.

### Peripheral immune cell contributions to AD progression and modulation of neuroinflammation

The central nervous system is considered an immune-privileged organ due to the blood-brain barrier (BBB) and the relatively low number of surveilling peripheral immune cells found within the brain parenchyma. Nonetheless, in the context of the chronic neuroinflammation that is present in AD, there is some evidence to suggest that peripheral immune cells infiltrate the CNS and accumulate near areas of pathology [94–96]. These peripheral immune cells include monocytes, which are highly phagocytic cells that migrate into areas of injury and proliferate and differentiate into macrophages to clear pathogens or cellular debris. Evidence suggests that inducing neuroinflammation in amyloidogenic mouse models by acute LPS injection or IL-1β overexpression leads to an increase of Ly6c+ CCR2+ monocyte migration into the central nervous system [97, 98]. Some studies have shown increased numbers of grafted monocytes associated with amyloid plaques in chimeric models and a dependence of amyloid clearance on the recruitment of CCR2+ cells [99–101]. Nonetheless, CCR2 knockout does not have an impact on IL-1β-induced clearance of amyloid β plaque by resident microglia and recent chimera studies that did not employ irradiation have shown very little evidence of migration [98]. Perhaps these monocytes could serve as a backup to resident microglial populations if the offending stimuli persist [102]. Interestingly, intracerebrally injected mesenchymal stem cells derived from the bone marrow or adipose tissue significantly reduced amyloid beta deposition and enhanced cognitive function, apparently by decreasing neuroinflammation [103, 104]. Thus, this pool of cells could be exploited to substitute for senescent microglia when they fail to slow AD progression [93, 105].

There is evidence to suggest migration of T lymphocytes into the brain of AD patients [106, 107]. As already noted, stimulation of microglia with amyloid β plaques in transgenic mouse models or injection of Aβ 1-42 oligomers lead to the release of proinflammatory cytokines such as IL-1β and TNFα. In vitro and in vivo studies have shown that these cytokines promote the production of chemokine CXCL8 by endothelial cells [108–111]. Interestingly, T cells of AD patients are enriched in the chemokine receptor CXCR2 which, upon binding to CXCL8, promotes T cell transmigration across endothelial barriers, including the blood-brain barrier [112]. This movement through the BBB was inhibited by anti-TNFα antibodies or inhibition of CXCR2 on T cells [112]. Other studies in rodent models have shown that once in the brain, T cells can modify microglia phenotype, increasing their motility and phagocytic activity through secretion of IFN γ [113]. Potentially, microglia could phagocytose and present amyloid β to T cells as a form of re-stimulation [114]. Whether this mechanism occurs in humans with AD and to what extent it is utilized remains an open question. Interestingly, *RAG1* and *RAG2* knockout mice, which lack B and T lymphocytes, demonstrated worse cognitive capabilities when compared to wild-type mice and increased amyloid β plaque deposition and microglia activation in amyloidogenic models [115, 116]. Nonetheless, these approaches have not yet teased out the specific contributions of different lymphocyte subtypes, which might explain differing results in some cases [117].

There are several types of T cells; among them are the classically defined CD8+ or cytotoxic T lymphocytes and CD4+ or helper T lymphocytes. The function of CD8 T cells is to infiltrate tissue and secrete enzymes such as perforin, granzymes, and granulysins that promote apoptosis of damaged or infected cells [118]. These cells can be found in AD patient brain tissue [96] and were shown in the hippocampus of tauopathy mouse models [119]. Indeed, inhibition of T cell infiltration was correlated with decreased microglial activation and cognitive impairment, without a significant impact on tau pathology [119].

In contrast to findings with cytotoxic T cells, T helper cells have been much more closely implicated in the response to AD pathology [116]. Th1 cells are responsible for promoting cellular responses to offending stimuli by activating macrophages and CD8+ T cells and their effector cytokine is IFNγ [120]. Contrastingly, Th2 cells are responsible for promoting humoral immune responses by inducing antibody production by B lymphocytes and some of their effector molecules are the anti-inflammatory cytokines IL-4 and IL-10, which inhibit Th1 responses [120]. In the context of AD, T helper cells have been shown to modulate microglial responses through contact or release of cytokines. Research into Th1 cells and IFNγ demonstrates that these cells can migrate into the brain parenchyma after immunization with Aβ 1-42 [95, 121, 122]. What they do once in the CNS is the subject of controversy. Some have argued that Th1 cells activate microglia to increase amyloid β clearance [113, 123], while others have claimed they increase pathology through IFNγ release [124]. This microglial activation also makes their processes more motile, which might help microglia encounter and surround amyloid β plaques more efficiently [124]. Interestingly, contrary to other proinflammatory cytokines, such as TNFα and IL-1β, overexpression of IFNγ using an AAV vector led to decreased tau pathology and increased neurogenesis [123]. However, the immune response of Th1 cells has been closely associated with the onset of meningoencephalitis in human subjects injected with an Aβ 1-42 vaccine, providing clear evidence that these cells increase proinflammatory responses [121, 125]. In contrast, induced Th2 cells may modulate glial responses with limited infiltration of the CNS, instead of acting from the choroid plexus or meningeal spaces [126–129]. Rodent studies have shown that when amyloid β specific Th2 cells are injected into the periphery, they modulate the cytokine profile of transgenic mice towards a more anti-inflammatory one by decreasing GM-CSF, TNFα, and IL-2 levels [128]. This peripheral modulation has been correlated with decreased microgliosis that is accompanied by improved cognitive performance [128]. Furthermore, Th2 cell activity might induce the promotion of Aβ 1-42 autoantibodies [130].

Attempts to harness the activity of the peripheral immune system to develop therapeutics for AD have been an ongoing subject of research. Active immunization with amyloid β was previously tried to promote clearance of AD pathology. While there is evidence that this therapy was able to improve cognition in some patients, this approach led to the development of meningoencephalitis in 6% of the subjects [131]. Since then, passive immunization approaches utilizing humanized antibodies against amyloid β, such as bapineuzumab and solanezumab, were developed and have since been tested in clinical trials [132, 133]. Many of these trials have concluded and most of the results are disappointing [134]. Ongoing trials are attempting to address whether treatment of individuals at risk at presymptomatic stages or utilizing higher doses can improve primary outcomes [134]. However, some have questioned whether targeting amyloid β production is sufficient to treat AD and if a revision of the amyloid hypothesis is needed [135, 136]. There has been a push to develop antibodies against tau oligomers, which are also known to be proinflammatory and may contribute to the spread of tau pathology [137]. Murine studies testing such approaches have succeeded in decreasing tau hyperphosphorylation after anti-tau oligomer administration [138, 139]. Currently, a study testing the humanized tau antibody ABBV-8E12 is enrolling patients in a phase II clinical trial [140].

In addition, others have proposed the use of the relapsing-remitting multiple sclerosis drug Copaxone or glatiramer acetate (GA) to treat AD [141–143]. GA is a mixture of peptides composed of the four amino acids found in myelin basic protein: glutamic acid, lysine, alanine, and tyrosine. While the mechanism of action of GA is still debated, evidence suggests that it is a stimulator of the Th2 response, which is beneficial in combating relapsing-remitting multiple sclerosis, possibly by suppressing the inflammatory, Th1 response [144–146]. Evaluation of serum and CNS of GA-treated mice showed increased levels of the anti-inflammatory cytokine IL-10 and brain-derived neurotrophic factor (BDNF), both of which are produced by activated Th2 cells [147–149]. In addition, not only has GA been effective in decreasing pathology in models of experimental autoimmune encephalomyelitis, but it has also shown benefits in Huntington’s disease models [149, 150]. GA use in amyloidogenic mouse models successfully reduced plaque load; however, the effects of GA on tau pathology remain untested [141–143].

The barriers that separate the periphery from the brain play an active role in regulating adaptive and innate immunity in the CNS. One of the most well-studied points of immune surveillance and trafficking into the CNS is the choroid plexus [151]. While leukocytes still have to receive chemoattractive signals to cross this structure, the fenestrated nature of the choroid plexus epithelium may be easier to traverse than the BBB. Recently, it has been shown that targeting the immune checkpoint protein programmed cell death 1 (PD-1) activates the choroid plexus epithelium and leads to an increase in monocyte trafficking through this structure, which hones to sites of amyloid deposition in the parenchyma [152]. This process is dependent on systemic T cell-derived IFNγ, most likely secreted by Th1 cells. Moreover, targeting immunosuppression mediated by regulatory T cells has a similar effect on immune trafficking through the choroid plexus in the 5xFAD model, which is correlated with improved cognition and pathology [142]. Nonetheless, it remains to be seen whether such approaches would be safe to consider long term and what effect they would have on neurofibrillary tangles. Uncontrolled T cell activity can not only increase hyperphosphorylation of tau but can also provoke more serious side effects such as encephalitis.

### Systemic inflammation and AD immunomodulation

Systemic infection and inflammation in individuals with AD is associated with worse cognitive function and reduced hippocampal volume [153–155]. Specifically, high serum levels of inflammatory cytokines like IL-1β and TNFα are associated with disease progression. In addition, some have shown that systemic infection is associated with an increased risk of developing AD [33, 156]. Induction of systemic disorders such as osteoarthritis in APP/PS1 mice led to glial activation and exacerbation of amyloid pathology [157]. After peripheral infection of APP/PS1 mice with the respiratory pathogen *Bordetella pertussis*, increased brain infiltration of IFNγ and IL-17 producing T cells and natural killer cells was observed that correlated with increased glial activation and amyloid β plaque deposition [158]. Both of these rodent studies highlight the interconnections between the peripheral inflammatory state and the CNS. It is clear that changes in concentrations of peripheral cytokines and infiltration of peripheral cells can have lasting effects on the response of microglia to AD pathology.

Microglia are incredibly sensitive to perturbations in the brain environment by peripheral stimuli including inflammation and infections. Several studies have utilized intraperitoneal injections of bacterial LPS to evaluate the effects of infection in AD [159–164]. In a recent study, peripheral LPS induced microglial activation and phenotype changes, including an increase in markers of disease-associated microglia, such as APOE and CLEC7A [88, 165]. LPS administration also increased accumulation of amyloid precursor protein and its cleavage product amyloid β early in disease [159, 166]. Another study found that this accumulation of amyloid β was exacerbated by an altered amyloid β efflux through the BBB in a low-density lipoprotein receptor-related protein 1 (LRP1)-dependent manner [166]. Interestingly, while a single LPS injection increased pro-inflammatory cytokines in the blood and brain [167], a persistent inflammatory tone established by sequential LPS injections can lead microglia to become more tolerant of inflammatory stimuli and dampen their response [165]. This last study also found that mice that had tolerant microglia also had lower levels of cerebral amyloidosis.

Modulation of pro-inflammatory and anti-inflammatory signals in the periphery has been explored as a potential target to ameliorate AD pathology. Blockade of the classic proinflammatory cytokine TNFα decreased amyloid β [168, 169] and tau pathology [170]. Inhibition of TNFα production using the small molecule thalidomide or one of its derivatives improved cognition and CNS pathology in models of systemic disease [171, 172]. Likewise, modulation of TNFα by an orally administered isoindolin-1,3 dithione (IDT) caused a reduction in fibrillar amyloid and tau hyperphosphorylation in the 3xTg AD mouse [173]. Interestingly, IDT treatment was also associated with increased neutrophil infiltration while reducing TNFα expression in these cells. Lastly, TNFα receptor antibodies such as etanercept or infliximab have been tested in dementia rat models and showed beneficial effects on cognitive behavior and some evidence of decreased AD pathology [174]. A case report administering perispinal etanercept led to a rapid cognitive improvement [175]. Recently, a small randomized, placebo-control trial of subcutaneous etanercept for AD was completed. Researchers found that while there were no significant differences between placebo and treated patients, the study revealed interesting trends that might be worth following up on with a larger, more heterogeneous cohort [176]. In addition to TNFα, peripheral blockade of other proinflammatory cytokines such as IL-12 and IL-23 had similar effects on amyloid pathology and rodent behavior, indicating that there is more than one potential target for proinflammatory cytokine modulation that could be exploited [177].

As discussed earlier, sterile inflammation has been implicated in AD progression. One of the cytokines playing a protective role in sterile inflammation is IL-33 [178]. IL-33 levels are low in the brains of AD patients, and serum levels of soluble ST2, the IL-33 receptor, are high in the brains of patients with mild cognitive impairment [179, 180]. Intraperitoneal injection of IL-33 ameliorated synaptic impairment and amyloid pathology in APP/PS1 mice [180]. Moreover, plaque-adjacent microglia in IL-33-injected mice showed increased expression of CD68, indicating increased phagolysosomal activity. In addition, there was an increase in levels of the enzyme neprilysin, which can aid in amyloid degradation, and decreased evidence of neuroinflammation, with lower levels of the molecules IL-1β, IL-6, and NLRP3 in the cortices of APP/PS1 mice [180]. IL-33 could be negatively regulating TLR4 activity by competing for MyD88, the binding partner of ST2 [181]. In another study, IL-33 deficient mice were found to develop severe neurodegeneration late in life that is characterized by abnormal accumulation of tau [182].

Activation of TLR9 with a peripheral injection of CpG oligodeoxynucleotides led to a reduction in both amyloid β and tau pathology in murine models of AD and thus could be beneficial [183–185]. TLR9 has been shown to regulate autoimmune responses in models of systemic lupus erythematosus [186]; therefore, it is possible that TLR9 in particular may regulate the inflammatory response in the context of AD, promoting an environment that leads to tissue repair. Taken together, there are many examples showing that alteration of peripheral inflammation by infectious agents or injections of cytokines or TLR ligands can have profound effects on brain AD pathology. More studies are needed to define mechanisms of CNS immunomodulation by peripheral factors. Nonetheless, the identification of such modulators is a promising and exciting route for therapeutic discovery. A recent review article by Jeffrey Cummings provides a comprehensive summary of immune-related and other therapeutics currently in the AD drug development pipeline [187].

### Current evidence from traditional anti-inflammatory medications

Observational studies, such as case-control and incidence studies, have shown that regular use of non-selective NSAIDs is associated with a reduced relative risk of developing AD [188–191]. The majority of individuals followed in these observational studies suffered from an inflammatory condition, such as rheumatoid arthritis, which explains their long-term NSAID use. Thus, the cohorts observed in these studies only represent a proportion of the population at risk of developing AD. A review article by Imbimbo et al. [192] has delineated the following general trends of risk reduction found in observational studies: (1) risk was modified by the length of time NSAIDs were used with the lowest risk being associated with 2 years of use, while no effect was detected with 1 month of use; (2) the type of NSAID use, with non-aspirin NSAIDs having a higher risk reduction when compared to aspirin, an irreversible COX inhibitor; (3) NSAIDs that were found to be effective at reducing risk of AD also have been shown to decrease levels of Aβ-42 in murine models of AD such as ibuprofen, indomethacin, and diclofenac.

Unfortunately, randomized control trials (RCT) have found that the use of naproxen, diclofenac, or indomethacin, all of which have been shown to reduce AD risk in epidemiological studies, do not slow down the progression of AD when compared to placebo [192–194]. One of the challenges for these trials was the dropout rate of subjects due to gastrointestinal issues brought on by chronic NSAID use. In order to prevent side effects, some RCTs minimized the daily dose of NSAID administered to the treatment group, which might potentially explain the negative results [192]. Trials utilizing COX 2 selective NSAIDs like celecoxib and rofecoxib, which are less likely to cause gastrointestinal issues, but retain risks for deleterious cardiovascular events, have failed to demonstrate positive treatment benefits for AD or mild cognitive impairment [195, 196].

Another potential mechanism of action of NSAIDs is activation of the peroxisome proliferator-activated receptor gamma (PPARγ) [192, 197]. This receptor is a member of the nuclear factor family, and its activation regulates the transcriptional activation and repression of several genes. One of the cellular actions resulting from PPARγ activation is the decrease of inflammation by reducing the production of proinflammatory cytokines such as IL-1β, IL-6, and TNFα [197]. At high concentrations, some NSAIDs can serve as an agonist of this receptor. Indomethacin and ibuprofen can activate microglial PPARγ and reduce amyloid β induced release of proinflammatory cytokines [198]. Treatment of amyloidogenic murine models with pioglitazone, a direct agonist of PPARγ, reduces amyloid β 1-42 deposits in the hippocampus [199]. The TOMMORROW study was a multicenter trial created to evaluate pioglitazone as a preventative agent for the development of AD [200]. However, the study was recently halted due to inadequate treatment effect.

## Conclusion

The contribution of specific pro-inflammatory and anti-inflammatory factors in AD is not straightforward, especially since the evaluation of cognition, amyloid β pathology, and neurofibrillary tangles yields conflicting results in mouse models. Furthermore, translating rodent studies that have modulated expression of specific cytokines in the CNS is challenging. In addition, studies that have shown promise, such as the beneficial effects of pioglitazone in mouse models of AD, do not always prove effective in humans. Nonetheless, the immune response is deeply tied to the development of pathology, and with advancing technologies, we are able to more fully dissect the complexity of this response and the effector cells that carry it out. Our knowledge of how microglia and peripheral immune cells interact has proved invaluable in understanding how this delicate balance goes awry in disease. Immunomodulation in AD offers multiple, promising pathways of investigation that might lead to therapeutics that can prevent or halt the development of amyloid and tau pathology and cognitive decline.

## Abbreviations

AD: Alzheimer’s disease
APOE: Apolipoprotein E
Aβ: Amyloid β
BBB: Blood-brain barrier
BDNF: Brain-derived neurotrophic factor
CNS: Central nervous system
COX 1: Cyclooxygenase 1
COX 2: Cyclooxygenase 2
CSF: Cerebrospinal fluid
DAM: Disease-associated microglia
GA: Glatiramer acetate
IFNγ: Interferon γ
IL-10: Interleukin 10
IL-12: Interleukin 12
IL-13: Interleukin 13
IL-17: Interleukin 17
IL-18: Interleukin 18
IL-1RA: Interleukin-1 receptor antagonist
IL-1β: Interleukin 1β
IL-33: Interleukin 33
IL-4: Interleukin 4
IL-4Rα: Interleukin 4 receptor α
IL-6: Interleukin 6
iNOS: Nitric oxide synthase
LPS: Lipopolysaccharide
NLRP3: NACHT, LRR, and PYD domain-containing protein 3
NSAIDs: Non-steroidal anti-inflammatory drugs
PPARγ: Peroxisome proliferator-activated receptor γ
RCT: Randomized control trials
TGF-β1: Transforming growth factor β1
TGF-β2: Transforming growth factor β2
TGFβRII: Transforming growth factor β receptor 2
Th1: Type 1 T helper cells
Th2: Type 2 T helper cells
TLR: Toll-like receptor
TNFα: Tumor necrosis factor α
TREM2: Triggering receptor expressed on myeloid cells 2
